# Dominance analysis of competing protein assembly pathways

**DOI:** 10.1371/journal.pone.0281964

**Published:** 2023-02-24

**Authors:** Johannes Lankeit, Stefanie Förste, Sophia Rudorf

**Affiliations:** 1 Institute of Applied Mathematics, Leibniz University Hannover, Hannover, Germany; 2 Theory and Bio-Systems, Max Planck Institute of Colloids and Interfaces, Potsdam, Germany; 3 Institute of Cell Biology and Biophysics, Leibniz University Hannover, Hannover, Germany; Minnan Normal University, CHINA

## Abstract

Most proteins form complexes consisting of two or more subunits, where complex assembly can proceed *via* two competing pathways: co-translational assembly of a mature and a nascent subunit, and post-translational assembly by two mature protein subunits. Assembly pathway dominance, i.e., which of the two pathways is predominant under which conditions, is poorly understood. Here, we introduce a reaction-diffusion system that describes protein complex formation *via* post- and co-translational assembly and use it to analyze the dominance of both pathways. Special features of this new system are (i) spatially inhomogeneous sources of reacting species, (ii) a combination of diffusing and immobile species, and (iii) an asymmetric binding competition between the species. We study assembly pathway dominance for the spatially homogeneous system and find that the ratio of production rates of the two protein subunits determines the long-term pathway dominance. This result is independent of the binding rate constants for post- and co-translational assembly and implies that a system with an initial post-translational assembly dominance can eventually exhibit co-translational assembly dominance and *vice versa*. For exactly balanced production of both subunits, the assembly pathway dominance is determined by the steady state concentration of the subunit that can bind both nascent and mature partners. The introduced system of equations can be applied to describe general dynamics of assembly processes involving both diffusing and immobile components.

## Introduction

**Fig 1:**
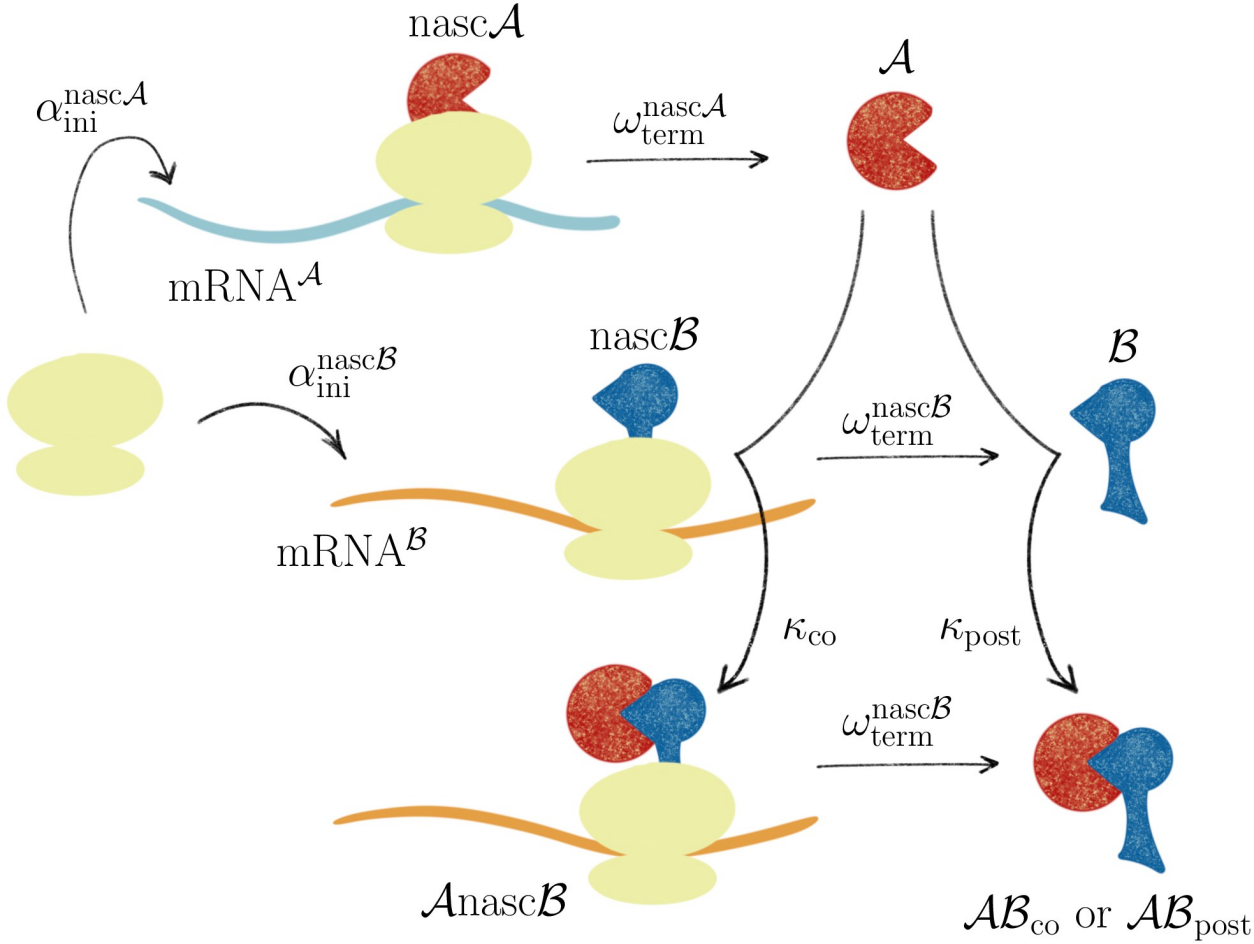
Post- and co-translational assembly of protein complexes. Ribosomes (yellow) bind to mRNA molecules (colored lines) with time- and position-dependent initiation rates $\alpha_{\text{ini}}^{\text{nasc}A}\left(\tilde{x},\tilde{t}\right)$ and $\alpha_{\text{ini}}^{\text{nasc}B}\left(\tilde{x},\tilde{t}\right)$, and synthesize protein subunits A and B with constant rates $\omega_{\text{term}}^{\text{nasc}A}$ and $\omega_{\text{term}}^{\text{nasc}B}$, respectively. Subunit A can bind subunit B co- or post-translationally with binding rate constants *κ*_co_ and *κ*_post_, respectively. In contrast, nascent subunit nascA cannot be bound by subunit B.

Proteins are peptide chains with lengths ranging from a few tens to ten thousands of amino acids. They constitute one of the most important classes of biomolecules as they are involved in all processes of life and fulfill a plethora of different tasks in living cells. The majority of proteins forms homo- or heterooligomers: multiple peptide chains assemble to form a functional protein complex [1], giving rise to all kinds of assemblies, from small antibodies to large structures like the tails of bacterial viruses. In cells, proteins are synthesized by biomolecular machines called ribosomes. Ribosomes use mRNA molecules as genetic templates to catalyze the sequential concatenation of individual amino acids into polypeptides. This process is called translation. During their synthesis, most nascent polypeptides fold into a defined threedimensional structure to become functional proteins when translation has finished and they are released from the ribosome. When proteins encounter each other, for example while diffusing in the cytosol or within the cell membrane, they can bind and form stable protein complexes. This is called *post-translational* assembly because complex formation occurs after the synthesis of the individual subunits. Shieh et al. [2] demonstrated that protein dimer assembly can also take place while one of the binding partners is still being synthesized by a ribosome. In this case, the mature binding partner (subunit A) binds to a part of the nascent chain of the second binding partner (subunit B) that is already exposed from the ribosome, see Fig 1. Protein A stays bound to nascent chain B until the latter is fully synthesized and remains bound afterwards. This complex formation pathway is called *co-translational* assembly. Co-translational assembly was shown to occur in bacteria [2] and yeast [3, 4], see [5] for a recent review, and was also proposed for an inner membrane protein [6] and a multiprotein complex [7]. In principle, both the post- and the co-translational assembly pathway lead to functional protein complexes. However, both pathways differ in a fundamental aspect: Co-translational assembly is asymmetric in the sense that subunit A can bind nascent subunit B but not *vice versa*, which implies that subunit A needs to be synthesized before subunit B. In contrast, for post-translational assembly the order of subunit synthesis is irrelevant. Furthermore, the nascent chain B is tethered to the translating ribosome and thus the encoding mRNA. Therefore, for co-translational assembly, binding partner A diffuses whereas the other component B is practically immobilized.

For the mathematical study of reactions, ordinary differential equations (ODEs) relating growth rates of concentrations with the amount of reactants by means of a mass action law have a long history [8–11]. Under fairly general conditions persistence and global stability properties, like convergence of all solutions to the appropriate steady state, have been shown (for convergence in case of an acyclic reaction graph for a closed system see [11, Sec. 4.2.2]; persistence for weakly reversible (or, more generally, lower endotactic) mass-action systems with bounded trajectories (under dimensionality conditions): [12]; proof of the global attractor conjecture for weakly reversible systems: [13]). Note, however, that not all reactions fulfil the conditions of these theorems (especially if they are non-conservative), and some may exhibit quite different properties; for an early example of periodic behavior in an ODE system for an autocatalytic reaction see [9].

Additionally including diffusion of each component leads to a system of parabolic partial differential equations. Such reaction-diffusion systems form another well-studied area of mathematics [14, 15], and it is known that inclusion of unequal diffusion rates may significantly affect the behavior of the system. For example, equilibria may be rendered unstable (Turing instabilities, see [16]), and physically reasonable assumptions (quasipositivity and mass dissipation) even no longer suffice for global existence of classical solutions, as the counterexample by Pierre and Schmitt [17] attests. Accordingly, such reaction-diffusion systems are interesting already on the level of existence theory of solutions and a rich mathematical literature has grown (see e.g., the survey [15] or [18–20] for some recent contributions), yielding, inter alia, bounded global classical solutions to reaction-diffusion systems with linear diffusion and at most slightly superquadratic reaction terms [19]. Systems with degenerate diffusion in the sense that only some of the chemical species diffuse are less frequently studied. At least for a certain single reversible reaction with one non-diffusible reagent (of, in total, two educts and two products), solutions, like in the non-degenerate case, exist globally and exponentially converge to the equilibrium (if the domain is spatially two-dimensional or if it is three-dimensional and the other diffusion rates sufficiently close to each other), as was recently shown in [21].

Here, we model protein heterodimer formation from two competing pathways as a reaction-diffusion system with diffusing and immobile components. We investigate under which conditions which of the two pathways is dominating the complex formation process. Due to the sustained production of the proteins, the system is nonconservative and permits unbounded solutions. Its notable features include immobility of one component, unboundedness of solutions, and competing reactions. Note that the quantity of interest is the relation between reaction rates, not the total concentrations of the reacting species.

## Results and discussion

### Molecular species and reaction parameters

We assume that the dynamics of protein heterodimer formation by post- and co-translational assembly depend on the following system parameters [22], see also Fig 1: The synthesis of nascent peptide chains nascA and nascB requires encountering and binding of corresponding mRNA molecules and ribosomes. In a cell, the distributions of mRNAs as well as ribosomes are non-uniform and time-dependent. Therefore, nascent chain synthesis is initiated with time- and position-dependent rates $\alpha_{\text{ini}}^{\text{nasc}A}\left(\tilde{x},\tilde{t}\right)$ and $\alpha_{\text{ini}}^{\text{nasc}B}\left(\tilde{x},\tilde{t}\right)$, respectively. Nascent chains are tethered to the translating ribosomes and, thus, immobilized until translation has terminated with constant rates $\omega_{\text{term}}^{\text{nasc}A}$ and $\omega_{\text{term}}^{\text{nasc}B}$, respectively. After translation has terminated, the nascent chains are released from the ribosomes and become free protein subunits A and B, respectively. A free subunit A can bind to a nascent chain nascB with binding rate constant *κ*_co_. The formed complex AnascB is immobile until the synthesis of subunit B is finished and the AnascB complex is released from the ribosome with rate $\omega_{\text{term}}^{\text{nasc}B}$. The released complex ${AB}_{\text{co}}$ is called *co-translationally* assembled complex to reflect its formation process. Alternatively, a free subunit A binds a free (released) subunit B with binding rate constant *κ*_post_ to form a *post-translationally* assembled complex ${AB}_{\text{post}}$. Free subunits as well as co- and post-translationally assembled complexes diffuse with diffusion constants $D_{A}$, $D_{B}$, and $D_{AB}$, respectively. With these system parameters, the time evolution of the concentrations $C_{}\left(\tilde{x},\tilde{t}\right)$ of nascent chains nascA and nascB, protein subunits A and B, nascent complex AnascB and mature complexes ${AB}_{\text{co}}$ and ${AB}_{\text{post}}$ is described by

$$\begin{array}{c} \frac{\partial }{\partial \tilde{t}}C_{\text{nasc}A}\left(\tilde{x},\tilde{t}\right)=\alpha_{\text{ini}}^{\text{nasc}A}\left(\tilde{x},\tilde{t}\right)-\omega_{\text{term}}^{\text{nasc}A}C_{\text{nasc}A}\left(\tilde{x},\tilde{t}\right) \end{array}$$ (1a)

$$\begin{array}{c} \frac{\partial }{\partial \tilde{t}}C_{\text{nasc}B}\left(\tilde{x},\tilde{t}\right)=\alpha_{\text{ini}}^{\text{nasc}B}\left(\tilde{x},\tilde{t}\right)-\omega_{\text{term}}^{\text{nasc}B}C_{\text{nasc}B}\left(\tilde{x},\tilde{t}\right)-\kappa_{\text{co}}C_{A}\left(\tilde{x},\tilde{t}\right)C_{\text{nasc}B}\left(\tilde{x},\tilde{t}\right) \end{array}$$ (1b)

$$\begin{array}{cc} \frac{\partial }{\partial \tilde{t}}C_{A}\left(\tilde{x},\tilde{t}\right) & =\omega_{\text{term}}^{\text{nasc}A}C_{\text{nasc}A}\left(\tilde{x},\tilde{t}\right)-\kappa_{\text{post}}C_{A}\left(\tilde{x},\tilde{t}\right)C_{B}\left(\tilde{x},\tilde{t}\right)-\kappa_{\text{co}}C_{A}\left(\tilde{x},\tilde{t}\right)C_{\text{nasc}B}\left(\tilde{x},\tilde{t}\right)\\ & \;+D_{A}\Delta C_{A}\left(\tilde{x},\tilde{t}\right) \end{array}$$ (1c)

$$\begin{array}{cc} \frac{\partial }{\partial \tilde{t}}C_{B}\left(\tilde{x},\tilde{t}\right) & =\omega_{\text{term}}^{\text{nasc}B}C_{\text{nasc}B}\left(\tilde{x},\tilde{t}\right)-\kappa_{\text{post}}C_{A}\left(\tilde{x},\tilde{t}\right)C_{B}\left(\tilde{x},\tilde{t}\right)\\ & \;+D_{B}\Delta C_{B}\left(\tilde{x},\tilde{t}\right) \end{array}$$ (1d)

$$\begin{array}{c} \frac{\partial }{\partial \tilde{t}}C_{A\text{nasc}B}\left(\tilde{x},\tilde{t}\right)=-\omega_{\text{term}}^{\text{nasc}B}C_{A\text{nasc}B}\left(\tilde{x},\tilde{t}\right)+\kappa_{\text{co}}C_{A}\left(\tilde{x},\tilde{t}\right)C_{\text{nasc}B}\left(\tilde{x},\tilde{t}\right) \end{array}$$ (1e)

$$\begin{array}{cc} \frac{\partial }{\partial \tilde{t}}C_{{AB}_{\text{co}}}\left(\tilde{x},\tilde{t}\right) & =\omega_{\text{term}}^{\text{nasc}B}C_{A\text{nasc}B}\left(\tilde{x},\tilde{t}\right)\\ & \;+D_{AB}\Delta C_{{AB}_{\text{co}}}\left(\tilde{x},\tilde{t}\right) \end{array}$$ (1f)

$$\begin{array}{cc} \frac{\partial }{\partial \tilde{t}}C_{{AB}_{\text{post}}}\left(\tilde{x},\tilde{t}\right) & =\kappa_{\text{post}}C_{A}\left(\tilde{x},\tilde{t}\right)C_{B}\left(\tilde{x},\tilde{t}\right)\\ & \;+D_{AB}\Delta C_{{AB}_{\text{post}}}\left(\tilde{x},\tilde{t}\right) \end{array}$$ (1g)

As usual, Δ denotes the Laplacian with respect to the spatial variable only.

**Remark 1**. *Some first observations show that* $C_{\text{nasc}A}$ *and* $C_{\text{nasc}B}$ *remain bounded as long as* $\alpha_{\text{ini}}^{\text{nasc}A}$ *and* $\alpha_{\text{ini}}^{\text{nasc}B}$ *are bounded functions. Furthermore*,

$$\begin{array}{c} \frac{d}{d\tilde{t}}\int (C_{\text{nasc}A}\left(\tilde{x},\tilde{t}\right)+C_{\text{nasc}B}\left(\tilde{x},\tilde{t}\right)+C_{A}\left(\tilde{x},\tilde{t}\right)+C_{B}\left(\tilde{x},\tilde{t}\right)\\ \;+2C_{A\text{nasc}B}\left(\tilde{x},\tilde{t}\right)+2C_{{AB}_{\text{co}}}\left(\tilde{x},\tilde{t}\right)+2C_{{AB}_{\text{post}}}\left(\tilde{x},\tilde{t}\right))d\tilde{x}\\ \;=\int \left(\alpha_{\text{ini}}^{\text{nasc}A}\left(\tilde{x},\tilde{t}\right)+\alpha_{\text{ini}}^{\text{nasc}B}\left(\tilde{x},\tilde{t}\right)\right)d\tilde{x}. \end{array}$$ (2)

For the sake of clarity, we make two simplifications to this reaction-diffusion system.

#### First simplifying assumption

The production of nascA is completely independent from all other reactions and we assume that is has reached its steady state at all positions $\tilde{x}$ in the system, which can easily be written explicitly. We assume that both the synthesis rate $\alpha_{\text{ini}}^{\text{nasc}A}$ and the concentration of nascent chains nascA are constant over time such that $\omega_{\text{term}}^{\text{nasc}A}C_{\text{nasc}A}\left(\tilde{x}\right)=\alpha_{\text{ini}}^{\text{nasc}A}\left(\tilde{x}\right)$ at steady state. Likewise, we assume that the synthesis of nascent chains nascB is constant over time such that the initiation rate $\alpha_{\text{ini}}^{\text{nasc}B}\left(\tilde{x}\right)$ is a function of space but not time.

**Remark 2**. *In this case*, (2) *immediately reveals that there is unlimited growth in the model as* $\tilde{t}\to \infty$, *which according to the first observation in Remark 1 has to take place in a component different from* $C_{\text{nasc}A}$ *and* $C_{\text{nasc}B}$. *On the other hand, it is not necessarily only the final products whose concentrations grow without bounds*:

$$\begin{array}{c} \frac{d}{d\tilde{t}}\int (C_{A}\left(\tilde{x},\tilde{t}\right)-(C_{\text{nasc}B}\left(\tilde{x},\tilde{t}\right)+C_{B}\left(\tilde{x},\tilde{t}\right)))\;d\tilde{x}=\int (\alpha_{\text{ini}}^{\text{nasc}A}\left(\tilde{x}\right)-\alpha_{\text{ini}}^{\text{nasc}B}\left(\tilde{x}\right))d\tilde{x}. \end{array}$$ (3)

*This already shows that the total amount of either* A *or* B *will tend to infinity, if* $\int (\alpha_{\text{ini}}^{\text{nasc}A}\left(\tilde{x}\right)-\alpha_{\text{ini}}^{\text{nasc}B}\left(\tilde{x}\right))d\tilde{x}$ *is positive or negative, respectively*.

#### Second simplification

In order to compare which of the two reaction paths is more important at each point in time and space, it makes more sense to compare $\frac{\partial }{\partial \tilde{t}}\left(C_{{AB}_{\text{co}}}\left(\tilde{x},\tilde{t}\right)+C_{A\text{nasc}B}\left(\tilde{x},\tilde{t}\right)\right)$ and $\frac{\partial }{\partial \tilde{t}}C_{{AB}_{\text{post}}}\left(\tilde{x},\tilde{t}\right)$ than $C_{{AB}_{\text{co}}}\left(\tilde{x},\tilde{t}\right)$ and $C_{{AB}_{\text{post}}}\left(\tilde{x},\tilde{t}\right)$. To this end, it is sufficient to know $C_{A}\left(\tilde{x},\tilde{t}\right)$, $C_{B}\left(\tilde{x},\tilde{t}\right)$, $C_{\text{nasc}B}\left(\tilde{x},\tilde{t}\right)$ and the coefficients, so that we may neglect the equations for $C_{{AB}_{\text{co}}}\left(\tilde{x},\tilde{t}\right)$, $C_{A\text{nasc}B}\left(\tilde{x},\tilde{t}\right)$, and $C_{{AB}_{\text{post}}}\left(\tilde{x},\tilde{t}\right)$ entirely.

If we additionally pick an arbitrary reference length *L*, duration $\tau =\frac{1}{\omega_{\text{term}}^{\text{nasc}B}}$, and abbreviate (and rescale) $x=\frac{1}{L}\tilde{x}$, $t=\frac{1}{\tau }\tilde{t}$,

$$\begin{array}{cc} a(x,t) & =\frac{\kappa_{\text{co}}}{\omega_{\text{term}}^{\text{nasc}B}}C_{A}\left(Lx,\frac{t}{\omega_{\text{term}}^{\text{nasc}B}}\right),\\ n(x,t) & =\frac{\kappa_{\text{co}}}{\omega_{\text{term}}^{\text{nasc}B}}C_{\text{nasc}B}\left(Lx,\frac{t}{\omega_{\text{term}}^{\text{nasc}B}}\right),\\ b(x,t) & =\frac{\kappa_{\text{co}}}{\omega_{\text{term}}^{\text{nasc}B}}C_{B}\left(Lx,\frac{t}{\omega_{\text{term}}^{\text{nasc}B}}\right), \end{array}$$

and set $d_{a}=\frac{D_{A}}{\omega_{term}^{\text{nasc}B}}\cdot \frac{1}{L^{2}}$, $d_{b}=\frac{D_{B}}{\omega_{term}^{\text{nasc}B}}\cdot \frac{1}{L^{2}}$, $\kappa_{a}\left(x\right)=\frac{\kappa_{co}\alpha_{ini}^{\text{nasc}A}\left(Lx\right)}{{\left(\omega_{term}^{\text{nasc}B}\right)}^{2}}$, $\kappa_{b}\left(x\right)=\frac{\kappa_{co}\alpha_{ini}^{\text{nasc}B}\left(Lx\right)}{{\left(\omega_{term}^{\text{nasc}B}\right)}^{2}}$ and $\gamma =\frac{\kappa_{post}}{\kappa_{co}}$, we finally end up with the following system of three equations:

$$\begin{array}{ccc} a_{t}=\kappa_{a}-an-\gamma ab & & +d_{a}\Delta a \end{array}$$ (4a)

$$\begin{array}{c} n_{t}=\kappa_{b}-n-an \end{array}$$ (4b)

$$\begin{array}{ccc} b_{t}=n-\gamma ab & & +d_{b}\Delta b \end{array}$$ (4c)

in Ω × (0, ∞), where we have written ${\left(\cdot \right)}_{t}=\frac{\partial }{\partial t}$ for the time derivative, $\Delta =\sum_{i=1}^{N}\frac{\partial^{2}}{\partial x_{i}^{2}}$, and where

$$\begin{array}{c} \Omega \subset R^{N},N\in N,\;\text{is}\;\text{a}\;\text{bounded}\;\text{domain}\;\text{with}\;\text{smooth}\;\text{boundary.} \end{array}$$ (5)

Note: While

$$\begin{array}{c} \gamma >0,d_{a}\geq 0,d_{b}\geq 0 \end{array}$$ (6)

are constant, *κ*_*a*_ and *κ*_*b*_ may depend on the spatial variable. They are, however, assumed to be nonnegative and constant w.r.t. time, see first simplification above. We will assume that

$$\begin{array}{c} \kappa_{a},\kappa_{b}\in C^{1}\left(\bar{\Omega }\right),\;\kappa_{a}\geq 0,\kappa_{b}\geq 0\;\;\text{in}\;\Omega . \end{array}$$ (7)

As long as diffusion is included in the description (that is, *d*_*a*_ or *d*_*b*_ are positive), we supplement (4) with homogeneous Neumann boundary conditions, where ∂_*ν*_ denotes the derivative in direction of the outward unit normal *ν*:

$$\begin{array}{c} d_{a}\partial_{\nu }a=0\;\;\text{in}\;\partial \Omega \times \left(0,\infty \right) \end{array}$$ (8a)

and

$$\begin{array}{c} d_{b}\partial_{\nu }b=0\;\;\text{in}\;\partial \Omega \times \left(0,\infty \right). \end{array}$$ (8b)

Additionally, initial data are prescribed:

$$\begin{array}{c} a\left(\cdot ,0\right)=a_{0},\;n\left(\cdot ,0\right)=n_{0},\;b\left(\cdot ,0\right)=b_{0}\;\text{in}\;\Omega , \end{array}$$ (9)

where we will assume that

$$\begin{array}{c} a_{0},n_{0},b_{0}\in C^{1}\left(\bar{\Omega }\right)\;\text{are}\;\text{nonnegative}\;\text{functions}. \end{array}$$ (10)

In the variables of (4), the rates with which the concentrations of protein complexes that can be attributed to the co-translational or post-translational assembly grow are given by *an* and *γab*, respectively.

### Solvability

In this brief section we will give a basic result on the full system (4). Since the main focus of the analytical investigations in this work will lie on the special case of *d*_*a*_ = *d*_*b*_ = 0, we keep the proof to a short outline.

**Theorem 3**. *We assume* (5), (6), (7) *and* (10).

*Then there is a unique global solution of* (4), (9), (8), *i.e. a triplet of functions* $\left(a,n,b\right)\in C(\bar{\Omega }\times \left[0,\infty \right))$ *such that* $a_{t},n_{t},b_{t},d_{a}\Delta a,d_{b}\Delta b\in C\left(\bar{\Omega }\times \left(0,\infty \right)\right)$ *and* (4), (9), (8) *are satisfied at each point*.

*This solution moreover satisfies*

$$\begin{array}{c} 0\leq a\left(x,t\right)\leq \|a_{0}{\|}_{L^{\infty }\left(\Omega \right)}+{\left\|\kappa_{a}\right\|}_{L^{\infty }\left(\Omega \right)}t\\ 0\leq n\left(x,t\right)\leq {\bar{c}}_{n}≔\text{max}\;\{\|n_{0}{\|}_{L^{\infty }\left(\Omega \right)},{\left\|\kappa_{b}\right\|}_{L^{\infty }\left(\Omega \right)}\}\\ 0\leq b\left(x,t\right)\leq \|b_{0}{\|}_{L^{\infty }\left(\Omega \right)}+{\bar{c}}_{n}t \end{array}$$

*for all* (*x*, *t*) ∈ Ω × (0, ∞).

*Proof*. The estimates can be obtained from comparison arguments. If *d*_*a*_ = *d*_*b*_ = 0, (4) is a system of ordinary differential equations (ODEs), and existence and uniqueness of a local solution are asserted by the Picard–Lindelöf theorem. That this solution is global follows from the bounds given above. For positive *d*_*a*_ and *d*_*b*_, we prove existence by employing a Schauder fixed point reasoning, which relies on general parabolic regularity (mainly [23, Theorems 14.4, 14.6, 15.5], [24, Theorem 4]) and (for Hölder regularity in the coupled PDE-ODE system) on a result like [25, Lemma 2.1]. Uniqueness is easily derived with the help of Grönwall’s inequality.

### The homogeneous case: *d*_*a*_ = *d*_*b*_ = 0, *a*_0_, *b*_0_, *n*_0_, *κ*_*a*_, *κ*_*b*_ constant

In this section, we investigate the system in the spatially homogeneous setting, finally giving a complete characterization of the long-term behavior of solutions with respect to the relative importance $\frac{\gamma ab}{an}$ of the reaction pathways.

In this simpler scenario, (4) is reduced to the ODE system

$$\begin{array}{c} a_{t}=\kappa_{a}-an-\gamma ab, \end{array}$$ (11a)

$$\begin{array}{c} n_{t}=\kappa_{b}-n-an, \end{array}$$ (11b)

$$\begin{array}{c} b_{t}=n-\gamma ab., \end{array}$$ (11c)

according to (9) and (10) supplemented with initial conditions

$$\begin{array}{c} a\left(0\right)=a_{0}\in \left[0,\infty \right),\;n\left(0\right)=n_{0}\in \left[0,\infty \right),\;b\left(0\right)=b_{0}\in \left[0,\infty \right). \end{array}$$ (11d)

A first general observation, irrespective of the size of the involved parameters, is the following conserved quantity:

$$\begin{array}{c} {\left(a-\left(n+b\right)\right)}_{t}=\kappa_{a}-\kappa_{b},\;\text{hence}\;a\left(t\right)=n\left(t\right)+b\left(t\right)+\left(\kappa_{a}-\kappa_{b}\right)t+c_{0}, \end{array}$$ (12)

where $c_{0}=a_{0}-n_{0}-b_{0}\in R$. Note that this corresponds to (3) for (1).

### The case of overproduction of B: *κ*_*a*_ < *κ*_*b*_

If there is an overproduction of B, the post-translational assembly pathway dominates: $\frac{\gamma ab}{an}\to \infty$, more precisely:

**Lemma 4**. *Let d*_*a*_ = *d*_*b*_ = 0 *and let a*_0_, *b*_0_, *n*_0_, *κ*_*a*_, *κ*_*b*_, *γ be positive constants with κ*_*a*_ < *κ*_*b*_. *Then the solution to* (11) *satisfies*

$$a\left(t\right)\to 0,\;b\left(t\right)\to \infty ,\;n\left(t\right)\to \kappa_{b},\;\;n_{t}\left(t\right)\to 0,\;b_{t}\left(t\right)-a_{t}\left(t\right)\to \kappa_{b}-\kappa_{a}\;\;\text{as}\;t\to \infty .$$

*Proof*. According to (12), *a* − (*n* + *b*) → −∞ as *t* → ∞, which, due to *a* ≥ 0, implies *n* + *b* → ∞. Given any *M* > 0, there is *T* > 0 such that for all *t* > *T* we have *n*(*t*) + *γb*(*t*) > *M* and thus

$$a_{t}\leq \kappa_{a}-Ma\;\text{on}\;\left(T,\infty \right),$$

which shows that ${\text{lim}\;\text{sup}}_{t\to \infty }\;a\left(t\right)\leq \frac{\kappa_{a}}{M}$. As *M* was arbitrary and *a* ≥ 0, therefore lim_*t*→∞_ *a*(*t*) = 0.

For every *ε* > 0, one can find *T* > 0 such that for *t* > *T*, *a*(*t*) < *ε*. For such *T*,

$$n_{t}=\kappa_{b}-n-an\geq \kappa_{b}-n-\varepsilon n\;\;\text{in}\;\left(T,\infty \right),$$

showing that ${\text{lim}\;\text{inf}}_{t\to \infty }n\left(t\right)\geq \frac{\kappa_{b}}{1+\varepsilon }$. As, additionally, lim sup_*t*→∞_ *n*(*t*) ≤ *κ*_*b*_ (because *n*_*t*_ ≤ *κ*_*b*_ − *n* on (0, ∞)), we obtain *n*(*t*) → *κ*_*b*_ as *t* → ∞.

As *n*(*t*) + *b*(*t*) → ∞ as *t* → ∞, this means that *b*(*t*) → ∞ as *t* → ∞.

Subsequently, can also conclude from (11) that *n*_*t*_ → 0 and *a*_*t*_ − *b*_*t*_ → *κ*_*a*_ − *κ*_*b*_.

### The case of overproduction of A: *κ*_*b*_ < *κ*_*a*_

If there is an overproduction of A, the concentrations of both nascB and B vanish in the large-time limit, as both are immediately used in reactions.

**Lemma 5**. *Let d*_*a*_ = *d*_*b*_ = 0 *and let a*_0_, *b*_0_, *n*_0_, *κ*_*a*_, *κ*_*b*_, *γ be positive constants with κ*_*a*_ > *κ*_*b*_. *Then the solution to* (11) *satisfies*

$$\begin{array}{c} a(t)\to \infty ,\;\;n(t)\to 0,\;\;b(t)\to 0\;\;\text{as}\;t\to \infty . \end{array}$$ (13)

*Proof*. By (12), *a* ≥ *a* − (*n* + *b*) → ∞ as *t* → ∞; in particular, *a*(*t*) → ∞ as *t* → ∞.

Let *M* > 0. Then there is *T* > 0 such that *a*(*t*) > *M* for all *t* > *T*. On (*T*, ∞), we have

$$n_{t}\leq \kappa_{b}-\left(M+1\right)n.$$

Therefore, by a comparison argument,

$$\underset{t\to \infty }{\text{lim}\;\text{sup}}\;n\left(t\right)\leq \frac{\kappa_{b}}{M+1}.$$

Employing this reasoning for arbitrarily large *M*, we obtain that lim_*t*→∞_ *n*(*t*) = 0.

Given *ε* > 0 and *M* > 0, there is *T* > 0 such that for every *t* > *T* we have *n*(*t*) < *ε* and $a\left(t\right)>\frac{M}{\gamma }$. Hence, on (*T*, ∞),

$$b_{t}=n-\gamma ab\leq \varepsilon -Mb,$$

so that ${\text{lim}\;\text{sup}}_{t\to \infty }b\left(t\right)\leq \frac{\varepsilon }{M}$, i.e. *b*(*t*) → 0 as *t* → ∞.

**Remark 6**. *According to Lemma 4 and Lemma 5, in both cases κ*_*a*_ < *κ*_*b*_ *and* *κ*_*a*_ > *κ*_*b*_, *the trajectories of the ODE system* (11) *are not persistent* (*cf*. [12, *Def. 2.12*]).

Although both concentrations *n* and *b* tend to 0, we can still reasonably ask which of the reaction pathways is stronger, that is how the quotient $\frac{b}{n}$ behaves. Even for large *γ*—i.e. when the binding rate constant for post-translational assembly exceeds that for co-translational assembly—it is almost immediately obtained from a study of $w=\frac{b}{n+b}$ that the co-translational pathway wins over the post-translational. As we will find in Lemma 8, for small *γ* > 0 the result is the same, although it is not as easily seen from the system.

**Lemma 7**. *In addition to the assumptions of Lemma 5 let γ* ≥ 1. *Then*

$$\frac{b(t)}{n(t)}\to 0\;\;\text{as}\;t\to \infty .$$

*Proof*. In order to see this, we introduce $w=\frac{b}{n+b}=\frac{\frac{b}{n}}{1+\frac{b}{n}}$ and show that *w*(*t*) → 0 as *t* → ∞. We conclude from (11) that

$$\begin{array}{c} w_{t}=1-w+\left(1-\gamma \right)aw\left(1-w\right)-\frac{\kappa_{b}}{n+b}w\;\text{in}\;\left(0,\infty \right). \end{array}$$ (14)

Since *γ* ≥ 1, the term (1 − *γ*)*aw*(1 − *w*) is negative so that (14) shows

$$w_{t}\leq 1-w-\frac{\kappa_{b}}{n+b}w\;\text{in}\;\left(0,\infty \right).$$

Due to (*κ*_*b*_ > 0 and) *n* + *b* → 0 (Lemma 5), given *M* > 0 we find *T* > 0 such that on (*T*, ∞) we have $\frac{\kappa_{b}}{n+b}+1>M$, that is

$$w_{t}\leq 1-Mw\;\;\text{on}\;\left(T,\infty \right),$$

i.e. ${\text{lim}\;\text{sup}}_{t\to \infty }w\left(t\right)\leq \frac{1}{M}$, hence lim_*t*→∞_ *w*(*t*) = 0.

**Lemma 8**. *In addition to the assumptions of Lemma 5 let γ* < 1. *Then*

$$\frac{b(t)}{n(t)}\to 0$$

*Proof*. We show this in two steps: Firstly, *ab* → 0 as *t* → ∞ (Lemma 9), secondly, *an* → *κ*_*b*_ as *t* → ∞ (Lemma 10), so that $\frac{b}{n}=\frac{ab}{an}\to 0$ as *t* → ∞.

**Lemma 9**. *Under the assumptions of Lemma 8*, *a*(*t*)*b*(*t*) → 0 *as* *t* → ∞.

*Proof*. Concerning the evolution of *ab*, system (11) implies

$${\left(ab\right)}_{t}=\kappa_{a}b-abn-\gamma ab^{2}+an-\gamma a^{2}b=\kappa_{a}b+a\left(-bn-\gamma b^{2}+n-\gamma ab\right).$$

Let us assume that lim sup_*t*→∞_ (*ab*)(*t*) ≥ *δ* for some *δ* > 0. Relying on (13) we choose *t*_1_ > 0 such that

$$\begin{array}{c} a>\frac{3}{\gamma },\;\kappa_{a}b<\frac{\delta }{2},\;-bn-\gamma b^{2}+n<\frac{\gamma \delta }{2}\;\text{on}\;\left[t_{1},\infty \right) \end{array}$$

and note that

$$\begin{array}{c} \text{if}\;t\geq t_{1}\;\text{and}\;\left(ab\right)\left(t\right)\geq \frac{3\delta }{4}\;\;\text{then}\;\\ {\left(ab\right)}_{t}\left(t\right)\leq \frac{\delta }{2}+a\left(\frac{\gamma \delta }{2}-\gamma ab\right)\leq \frac{\delta }{2}+a\gamma \left(\frac{\delta }{2}-\frac{3\delta }{4}\right)=\frac{\delta }{2}-\frac{a\gamma \delta }{4}\leq -\frac{\delta }{4} \end{array}$$ (15)

Furthermore, we let ${\left\{t_{2}^{\left(k\right)}\right\}}_{k\in N}$ be an increasing sequence with limit ∞ such that $ab\left(t_{2}^{\left(k\right)}\right)>\frac{3\delta }{4}$ for each k∈N, introduce

$$M_{k}≔\{t\in \left[t_{1},t_{2}^{\left(k\right)}\right]|\left(ab\right)\left(t\right)<\frac{3\delta }{4}\}$$

and assume that *M*_*k*_ ≠ ∅. Then $t_{3}^{\left(k\right)}=\text{sup}M_{k}\in \left[t_{1},t_{2}^{\left(k\right)}\right)$ is well-defined and

$$\left(ab\right)\left(t\right)\geq \frac{3\delta }{4}\;\text{for}\;\text{each}\;t\in \left(t_{3}^{\left(k\right)},t_{2}^{\left(k\right)}\right).$$

According to (15), ${\left(ab\right)}_{t}\left(t\right)\leq -\frac{\delta }{4}<0$ for these *t*, so that

$$\frac{3\delta }{4}<\left(ab\right)\left(t_{2}^{\left(k\right)}\right)\leq \left(ab\right)\left(t_{3}^{\left(k\right)}\right)\leq \frac{3\delta }{4},$$

a contradiction. Hence, *M*_*k*_ = ∅, that is

$$ab\geq \frac{3\delta }{4}\;\;\text{on}\;\underset{k\in N}{⋃}\left[t_{1},t_{2}^{\left(k\right)}\right)=\left[t_{1},\infty \right).$$

Again by (15), we therefore may conclude that ${\left(ab\right)}_{t}<-\frac{\delta }{4}$ on [*t*_1_, ∞), which implies (*ab*)(*t*) → −∞ as *t* → ∞, in contradiction to the nonnegativity of *ab*.

We conclude that lim sup_*t*→∞_ (*ab*)(*t*) = 0 and thus *ab* → 0.

Similar reasoning shows *an* → *κ*_*b*_:

**Lemma 10**. *Under the assumptions of Lemma 8*, *a*(*t*)*n*(*t*) → *κ*_*b*_ *as* *t* → ∞.

*Proof*. We assume that lim sup_*t*→∞_ *an* ≥ *κ*_*b*_ + *δ* for some *δ* > 0 and, aided by (13), let *t*_1_ > 0 be such that

$$\kappa_{a}n<\frac{\delta }{2},\;a>2\;\;\text{on}\;\left(t_{1},\infty \right).$$

Letting ${\left\{t_{2}^{\left(k\right)}\right\}}_{k\in N}$ be a monotone sequence with ${\text{lim}}_{k\to \infty }t_{2}^{\left(k\right)}=\infty$ such that

$$\left(an\right)\left(t_{2}^{\left(k\right)}\right)>\kappa_{b}+\frac{\delta }{2}\;\text{for}\;\text{each}\;k\in N,$$

we let $M_{k}=\{t\in \left[t_{1},t_{2}^{\left(k\right)}\right]|\left(an\right)\left(t\right)<\kappa_{b}+\frac{\delta }{2}\}$ and $t_{3}^{\left(k\right)}=\text{sup}M_{k}$. If we assume that *M*_*k*_ ≠ ∅, then $t_{3}^{\left(k\right)}$ exists and satisfies $t_{3}^{\left(k\right)}<t_{2}^{\left(k\right)}$ and for $t\in (t_{3}^{\left(k\right)},t_{2}^{\left(k\right)})$, we have $\left(an\right)\left(t\right)\geq \kappa_{b}+\frac{\delta }{2}$ according to the definition of $t_{3}^{\left(k\right)}$. This implies that

$$\begin{array}{cc} {\left(an\right)}_{t} & =\kappa_{a}n+a\left(-n^{2}-\gamma bn+\kappa_{b}-n-an\right)\leq \kappa_{a}n+a\left(\kappa_{b}-an\right)\\ & \leq \frac{\delta }{2}+a\left(\kappa_{b}-\left(\kappa_{b}+\frac{\delta }{2}\right)\right)=\frac{\delta }{2}\left(1-a\right)<-\frac{\delta }{2} \end{array}$$

on $\left(t_{3}^{\left(k\right)},t_{2}^{\left(k\right)}\right)$, in particular $\left(an\right)\left(t_{2}^{\left(k\right)}\right)<\left(an\right)\left(t_{3}^{\left(k\right)}\right)$, contradicting the definitions of $t_{2}^{\left(k\right)}$ and $t_{3}^{\left(k\right)}$. Therefore, *M*_*k*_ = ∅ for each k∈N and

$$an\geq \kappa_{b}+\frac{\delta }{2}\;\;\text{on}\;\underset{k\in N}{⋃}\left[t_{1},t_{2}^{\left(k\right)}\right)=\left[t_{1},\infty \right).$$

As above, this entails that ${\left(an\right)}_{t}<-\frac{\delta }{2}$ on (*t*_1_, ∞), which in turn proves *an* → −∞, in contradiction to the nonnegativity of *a* and *n*. In conclusion, lim sup_*t*→∞_ (*an*) ≤ *κ*_*b*_.

Now we assume liminf_*t*→∞_ (*an*) ≤ *κ*_*b*_ − *δ* for some *δ* > 0. With *t*_1_ > 0 chosen such that *a* > 1 and $n^{2}+\gamma nb+n<\frac{\delta }{4}$ on (*t*_1_, ∞), we have

$${\left(an\right)}_{t}=\kappa_{a}n+a\left(-n^{2}-\gamma bn-n+\kappa_{b}-an\right)\geq a\left(-\frac{\delta }{4}+\kappa_{b}-an\right)\;\;\text{on}\;\left(t_{1},\infty \right).$$

If ${\left\{t_{2}^{\left(k\right)}\right\}}_{k\in N}$ is, again, a monotone increasing divergent sequence such that $\left(an\right)\left(t_{2}^{\left(k\right)}\right)<\kappa_{b}-\frac{\delta }{2}$ for every k∈N, $M_{k}=\{t\in \left[t_{1},t_{2}^{\left(k\right)}\right]|\left(an\right)\left(t\right)>\kappa_{b}-\frac{\delta }{2}\}$ and—under the assumption that *M*_*k*_ be nonempty—$t_{3}^{\left(k\right)}=\text{sup}M_{k}$, we see that $an\leq \kappa_{b}-\frac{\delta }{2}$ on $\left(t_{3}^{\left(k\right)},t_{2}^{\left(k\right)}\right)$, and thus

$${\left(an\right)}_{t}\geq a\left(-\frac{\delta }{4}+\kappa_{b}-an\right)\geq a\frac{\delta }{4}\geq \frac{\delta }{4}$$

on $\left(t_{3}^{\left(k\right)},t_{2}^{\left(k\right)}\right)$. As consequence, $\left(an\right)\left(t_{2}^{\left(k\right)}\right)\geq \left(an\right)\left(t_{3}^{\left(k\right)}\right)$, contradicting the definitions of $t_{2}^{\left(k\right)}$ and $t_{3}^{\left(k\right)}$. Thus, *M*_*k*_ = ∅ and

$$an\leq \kappa_{b}-\frac{\delta }{2}\;\text{on}\;\left(t_{1},\infty \right).$$

Therefore ${\left(an\right)}_{t}\geq \frac{\delta }{4}$ on (*t*_1_, ∞), so that (*an*)(*t*) → ∞ as *t* → ∞, which contradicts lim sup_*t*→∞_ (*an*)(*t*) ≤ *κ*_*b*_ as well as the assumption liminf_*t*→∞_ *an* ≤ *κ*_*b*_ − *δ*. In conclusion, liminf_*t*→∞_ (*an*)(*t*) ≥ *κ*_*b*_. Together with the first part, this shows lim_*t*→∞_ (*an*)(*t*) = *κ*_*b*_.

### The special case of balanced production *κ*_*a*_ = *κ*_*b*_

In the previous two subsections we have seen that if the production of either A or B exceeds that of the other, this component accumulates in the system and determines which of the reaction pathways is more important on long time scales. We will now, in contrast, consider the case where the production rates of nascA and nascB are precisely in balance: *κ*_*a*_ = *κ*_*b*_.

While it can be argued that exact equality of parameters is never found in reality, this case is interesting as the critical case where the system behavior is not determined by oversaturation with one of the two proteins. (Taking into account that the assumption of time-independence of the parameters already is an approximation that hides fluctuations, equality of these parameters can on the other hand be seen as *the* relevant and most appropriate choice among constants for all scenarios where there is no unlimited buildup of any of the two components in the long term.)

In this case we set

$$\kappa ≔\kappa_{a}=\kappa_{b}$$

and first observe that any surplus of one of the protein types is conserved for all times:

$$\begin{array}{c} a-\left(n+b\right)=c_{0}=a_{0}-n_{0}-b_{0}\in R\;\;\text{on}\;\left(0,\infty \right) \end{array}$$ (16)

This allows us to write (11) equivalently as

$$\begin{array}{c} a_{t}=\kappa -\gamma a^{2}+\gamma c_{0}a+\left(\gamma -1\right)an \end{array}$$ (17a)

$$\begin{array}{c} n_{t}=\kappa -n-an \end{array}$$ (17b)

or

$$\begin{array}{c} a_{t}=\kappa -a^{2}+ac_{0}+\left(1-\gamma \right)ab \end{array}$$ (18a)

$$\begin{array}{c} b_{t}=-b-c_{0}+a-\gamma ab \end{array}$$ (18b)

or

$$\begin{array}{c} n_{t}=\kappa -n-n^{2}-nc_{0}-nb \end{array}$$ (19a)

$$\begin{array}{c} b_{t}=-\gamma b^{2}-\gamma c_{0}b+n-\gamma bn. \end{array}$$ (19b)

We can already note a first difference to the earlier cases where one of the concentrations grew without bounds:

**Lemma 11**. *Let d*_*a*_ = *d*_*b*_ = 0 *and let a*_0_, *b*_0_, *n*_0_, *γ* *be positive constants and κ* = *κ*_*a*_ = *κ*_*b*_ > 0. *Then there are constants* ${\underline{c}}_{a},{\bar{c}}_{a},{\underline{c}}_{n},{\bar{c}}_{n},{\underline{c}}_{b},{\bar{c}}_{b}>0$ *such that the solution* (*a*, *n*, *b*) *to* (11) *satisfies*

$${\underline{c}}_{a}<a\left(t\right)<{\bar{c}}_{a},\;{\underline{c}}_{n}<n\left(t\right)<{\bar{c}}_{n},\;{\underline{c}}_{b}<b\left(t\right)<{\bar{c}}_{b}$$

*for all t* ∈ (0, ∞). *Moreover*,

$$\begin{array}{c} \underset{t\to \infty }{\text{lim}\;\text{inf}}\;a\left(t\right)\geq c_{0} \end{array}$$ (20)

*with c*_0_ *as in* (16).

*Proof*. From (17b) boundedness of *n* from above is immediate and subsequently (19b) and (18a) make boundedness of *b* and *a*, respectively, obvious. Using boundedness of *a* and (17b), we also find a positive lower bound for *n*; (18a) and $b\leq {\bar{c}}_{b}$ entail a lower bound ${\underline{c}}_{a}>0$ of *a*, whereas ${\bar{c}}_{n}\geq n\geq {\underline{c}}_{n}$ and (19b) yield a positive lower bound for *b*.

As to (20), we know from (11), nonnegativity of *n* and *b* and (16) that

$$a_{t}=\kappa -an-\gamma ab\geq \kappa -a\;\text{max}\{1,\gamma \}\left(n+b\right)=\kappa -a\;\text{max}\{1,\gamma \}\left(a-c_{0}\right).$$

Since the solution $\underline{a}$ of

$${\underline{a}}_{t}=\kappa -\underline{a}\;\text{max}\{1,\gamma \}\left(\underline{a}-c_{0}\right),\;\underline{a}\left(0\right)=a_{0}$$

satisfies ${\text{lim}}_{t\to \infty }\underline{a}\left(t\right)={\underline{a}}_{\infty }$, where

$${\underline{a}}_{\infty }=\frac{c_{0}+\sqrt{c_{0}^{2}+4\frac{\kappa }{\text{max}\{1,\gamma \}}}}{2},$$

we conclude (from a comparison argument) that ${\text{lim}\;\text{inf}}_{t\to \infty }a\left(t\right)\geq {\underline{a}}_{\infty }>c_{0}$.

Lemma 11 shows that the relative importance $\frac{\gamma ab}{an}$ of the reaction pathways remains bounded between positive constants.

**Lemma 12**. *Let d*_*a*_ = *d*_*b*_ = 0 *and let a*_0_, *b*_0_, *n*_0_, *γ* *be positive constants and κ* = *κ*_*a*_ = *κ*_*b*_ > 0. *If the solution* (*a*, *n*, *b*) *of* (11) *converges as t* → ∞, *then*

$$\underset{t\to \infty }{\text{lim}}a\left(t\right)=a_{\infty },\;\underset{t\to \infty }{\text{lim}}n\left(t\right)=\frac{\kappa }{a_{\infty }+1},\;\underset{t\to \infty }{\text{lim}}b\left(t\right)=\frac{\kappa }{\gamma a_{\infty }\left(a_{\infty }+1\right)},$$

*where a*_∞_ *is the unique positive solution of*

$$\begin{array}{c} p\left(a\right)=a^{3}+\left(1-c_{0}\right)a^{2}-\left(\kappa +c_{0}\right)a-\frac{\kappa }{\gamma }=0 \end{array}$$ (21)

*with c*_0_ *as in* (16).

*Proof*. The only possible limits for convergent solutions of ODEs are the steady states. Given $c_{0}\in R$, all steady states fulfilling (16) are characterized by the equations given in this lemma. Among the roots of *p* exactly one is positive, and according to Lemma 11 this is the only solution of (21) that could be a limit of *a*.

**Theorem 13**. *Let d*_*a*_ = *d*_*b*_ = 0 *and let a*_0_, *b*_0_, *n*_0_, *γ* *be positive constants and κ* = *κ*_*a*_ = *κ*_*b*_ > 0. *Then the solution* (*a*, *n*, *b*) of (11) *converges, and*

$$\underset{t\to \infty }{\text{lim}}\frac{an}{\gamma ab}=a_{\infty }$$

*with a*_∞_ *being the root of* (21), *which is monotone increasing with respect to κ* *and c*_0_ = *a*_0_ − *n*_0_ − *b*_0_ *and decreasing with respect to γ*.

*Proof*. All possible limits of convergent solutions have been identified in Lemma 12. It remains to show that all solutions actually converge. We treat different ranges of values of *γ* and *c*_0_ separately.

**Case I**: If *γ* ≤ 1, (17) is a competitive (two-dimensional) system. All of its bounded solutions (hence, by Lemma 17: all solutions) therefore converge, see [26].

**Case II**: *γ* > 1, $c_{0}>-\frac{1}{\gamma }$: We cover this case with the following Lyapunov type reasoning: Starting from (18), for arbitrary *B* > 0 we compute

$$\begin{array}{c} \frac{d}{dt}(\frac{1}{2}{\left(a-a_{\infty }\right)}^{2}+\frac{B}{2}{\left(b-b_{\infty }\right)}^{2})=\\ -{\left(a-a_{\infty }\right)}^{2}(a_{\infty }+a-c_{0}+\left(\gamma -1\right)b)\\ -{\left(b-b_{\infty }\right)}^{2}(B+B\gamma a)+\left(a-a_{\infty }\right)\left(b-b_{\infty }\right)(\left(1-\gamma \right)a_{\infty }+B-B\gamma b_{\infty }) \end{array}$$

where *a*_∞_ is taken from Lemma 12 and $b_{\infty }=\frac{a_{\infty }-c_{0}}{1+\gamma a_{\infty }}$ so that 0 = −*b*_∞_ − *c*_0_ + *a*_∞_ − *γa*_∞_*b*_∞_ and $0=\kappa -a_{\infty }^{2}+a_{\infty }c_{0}+\left(1-\gamma \right)a_{\infty }b_{\infty }$. We note that $c_{0}>-\frac{1}{\gamma }$ implies $b_{\infty }<\frac{1}{\gamma }$ and hence $B=\frac{\left(\gamma -1\right)a_{\infty }}{1-\gamma b_{\infty }}$ is positive and satisfies (1 − *γ*)*a*_∞_ + *B* − *Bγb*_∞_ = 0.

By Lemma 11, there are *T* > 0 and *δ* > 0 such that 2(*a*_∞_ + *a*(*t*) − *c*_0_ + (*γ* − 1)*b*(*t*)) ≥ *δ* and $\frac{2}{B}\left(B+B\gamma a\left(t\right)\right)>\delta$ for all *t* ≥ *T*. Therefore, $V\left(t\right)=\frac{1}{2}{\left(a\left(t\right)-a_{\infty }\right)}^{2}+\frac{B}{2}{\left(b\left(t\right)-b_{\infty }\right)}^{2}$ satisfies

$$V^{\prime }\left(t\right)\leq -\delta V\left(t\right)\;\text{for}\;\text{all}\;t\geq T,$$

showing that *a*(*t*) → *a*_∞_ and *b*(*t*) → *b*_∞_ as *t* → ∞.

**Case IIIa**: *γ* > 1, $c_{0}\leq -\frac{1}{\gamma }$: We assume there is some *t*_0_ ≥ 0 such that $b\left(t_{0}\right)\geq \frac{1}{\gamma }$. We note that $\underline{b}=\frac{1}{\gamma }$ is a subsolution to (19b):

$${\underline{b}}_{t}=0\leq -\frac{1}{\gamma }-c_{0}+n-n=-\gamma {\underline{b}}^{2}-\gamma c_{0}\underline{b}+n-\gamma \underline{b}n,$$

because $c_{0}\leq -\frac{1}{\gamma }$. Therefore, by a comparison argument, $b\left(t\right)\geq \frac{1}{\gamma }$ for all *t* > *t*_0_. On the set $\left\{\left(n,b\right)\in R^{2}|n\geq 0,b\geq \frac{1}{\gamma }\right\}$, system (19) is competitive, and hence its solutions converge according to Hirsch’s result on two-dimensional cooperative and competitive systems of ODEs [26].

**Case IIIb**: *γ* > 1, $c_{0}\leq -\frac{1}{\gamma }$: Now we assume $b\left(t\right)<\frac{1}{\gamma }$ for all *t* > 0. In this case, according to (18b),

$$b_{t}=-b-c_{0}+a-\gamma ab\geq -b-c_{0}+a-\gamma a\frac{1}{\gamma }=-b-c_{0}\;\text{for}\;\text{all}\;t>0$$

and therefore

$$\underset{t\to \infty }{\text{lim}\;\text{inf}}b\left(t\right)\geq -c_{0}.$$

If $c_{0}<-\frac{1}{\gamma }$, this contradicts the assumption $b\left(t\right)<\frac{1}{\gamma }$ for all times; if $c_{0}=-\frac{1}{\gamma }$, then ${\text{lim}}_{t\to \infty }b\left(t\right)=\frac{1}{\gamma }$ and convergence of *n* or *a* is easily obtained from (18a) or (19a).

## Conclusion

Here, we introduced the reaction-diffusion system (1) and its simplified version (2) to describe the time-evolution of protein complex assembly via two competing pathways: co-translational assembly of a mature subunit A and a nascent subunit nascB, and post-translational assembly by mature protein subunit A and mature protein subunit B forming a protein complex.

Special features of this new system are (i) the presence of terms for spatially inhomogeneous sources of reacting species A and nascB, (ii) the combination of diffusing species A and B and immobile species nascB, and (iii) the asymmetric competition for reaction between diffusing and immobile species. We proved existence and uniqueness of solutions of the spatially inhomogeneous system and characterized the long-term behavior for the spatially homogeneous system. In our analysis, we were not particularly interested in the limit values of the concentrations of the system components. Instead, we studied the ratio of the post- and co-translational assembly reaction rates, i.e., the assembly pathway dominance. We found that an overproduction of subunit B, which can only bind mature partners A, leads to a long-term dominance of post-translational assembly. In contrast, overproduction of subunit A, which can bind both nascent and mature partners nascB and B, eventually leads to dominance of the co-translational assembly pathway. Note that these results are independent of the binding rate constants for post- and co-translational assembly. This implies that a system with an initial post-translational assembly dominance and overproduction of subunit A eventually shows co-translational assembly dominance and, *vice versa*, assembly is post-translationally dominated in the long term in a system with an initial co-translational assembly dominance and overproduction of subunit B. For exactly balanced production of both species A and nascB, the relative importance of the co- and post-translational assembly pathways remains bounded and its long-term value is determined by the steady state concentration of subunit A.

Further analyses of the system are needed to investigate the influence of spatially inhomogeneous synthesis of species A and nascB on the relative pathway dominance. Of course, our results are general in the sense that they do not only apply to protein complex assembly but to any assembly system comprising both diffusing and immobile components.
